# Association between door-to-wire time and 30-day mortality after PCI in patients with acute myocardial infarction: evidence from a single-center study in the China chest pain center registry

**DOI:** 10.3389/fcvm.2026.1717258

**Published:** 2026-02-18

**Authors:** Yongguang Wang, Te Xu, Yunrui Zhang, Fanqi Kong, Yuzhan Lin

**Affiliations:** 1 Department of Cardiovascular Medicine, The Third Affiliated Hospital of Wenzhou Medical University, Ruian, Zhejiang, China; 2 Department of Clinical Laboratory, The Third Affiliated Hospital of Wenzhou Medical University, Ruian, Zhejiang, China

**Keywords:** acute myocardial infarction, complications, door-to-wire time, mortality, percutaneous coronary intervention, restricted cubic spline

## Abstract

**Background:**

Door-to-wire (D2W) time may reflect the initiation of mechanical reperfusion in Acute myocardial infarction (AMI) more precisely. However, evidence on its association with short-term mortality remains limited, and the potential nonlinear or threshold relationship between D2W time and mortality has not been well characterized. This study aims to evaluate the association between D2W time and in-hospital mortality within 30 days and to explore potential nonlinear relationships.

**Methods:**

In this retrospective cohort study, we included patients with AMI who underwent percutaneous coronary intervention (PCI) at a certified Chest Pain Center in China between January 2021 and April 2025. The primary outcome was in-hospital mortality within 30 days of admission, and secondary outcomes included intraoperative and in-hospital complications. D2W time was analyzed as a continuous exposure (per 10-min increase). Multivariable Cox proportional hazards models with restricted cubic splines (RCS) were used to characterize potential nonlinear associations between D2W time and outcomes. RCS analyses were repeated in prespecified subgroups to assess robustness.

**Results:**

Among 1,451 AMI patients who underwent PCI, longer D2W time was associated with a higher risk of in-hospital mortality within 30 days of admission in the fully adjusted Cox model (per 10-min increase: HR 1.47, 95% CI 1.16–1.87; *P* = 0.0016). RCS analyses suggested a nonlinear association, with the fitted curve reaching its lowest point at approximately 50 min and increasing thereafter. Longer D2W times were also associated with higher risks of in-hospital infection and respiratory failure. Findings were broadly consistent across prespecified subgroup analyses.

**Conclusion:**

Among patients with AMI undergoing PCI, longer D2W time was associated with higher risks of 30-day in-hospital mortality and in-hospital complications. RCS analyses suggested a nonlinear association between D2W time and 30-day in-hospital mortality, and further validation in future studies is needed.

## Introduction

Acute myocardial infarction (AMI) remains a leading cause of morbidity and mortality from cardiovascular disease worldwide, with an estimated annual incidence of 15.9 million cases (1). For patients with ST-segment elevation myocardial infarction (STEMI) and high-risk non–ST-segment elevation myocardial infarction (NSTEMI), primary percutaneous coronary intervention (PCI) has been established as the gold-standard reperfusion therapy, offering superior efficacy to thrombolysis when performed in a timely manner (2, 3).

The concept that “time is muscle” has driven the development of quality metrics aimed at optimizing the timeliness of reperfusion therapy. Although door-to-balloon (D2B) time (4) has traditionally served as the primary performance indicator, emerging evidence suggests that door-to-wire (D2W) time (5) may provide a more precise measure of treatment delay, as it reflects the actual initiation of mechanical reperfusion. D2W is defined as the interval from hospital arrival to the passage of the guidewire across the culprit lesion, thereby excluding the additional time required for balloon inflation and stent deployment.

The European Society of Cardiology (ESC) guidelines emphasize minimizing system delay and use the interval from STEMI diagnosis (ECG interpreted as STEMI) to PCI-mediated reperfusion (guidewire crossing) as a key performance target for primary PCI (6). In routine practice, the time from hospital arrival to guidewire crossing (door-to-wire time, D2W) is also a readily captured, workflow-oriented metric that reflects in-hospital process efficiency. Although growing evidence suggests that shorter treatment delays are associated with improved outcomes in AMI patients undergoing PCI (5, 7), the nonlinear relationship between D2W time and clinical outcomes remains incompletely defined. In China, the establishment of chest pain centers has markedly improved the quality of AMI care by implementing standardized pathways for rapid diagnosis and treatment (8). The China Chest Pain Center Registry has provided a valuable platform for evaluating clinical outcomes and quality indicators in AMI management. However, data regarding the specific association between D2W time and mortality among Chinese patients with AMI are still limited.

Accordingly, the primary objective of this study was to investigate the association between D2W time and in-hospital mortality within 30 days of admission among patients with AMI undergoing PCI, using data from a single-center cohort within the China Chest Pain Center Registry. We further aimed to characterize the dose–response relationship and assess potential nonlinearity. We hypothesized that prolonged D2W time would be associated with a higher risk of 30-day in-hospital mortality and that this association might be non-linear.

## Methods

### Data sources and setting

This retrospective cohort study was conducted at the Third Affiliated Hospital of Wenzhou Medical University, a tertiary care center and certified chest pain center participating in the China Chest Pain Center Registry. The Chest Pain Center initiative in China aims to standardize regional pathways for rapid diagnosis, triage, and reperfusion for acute chest pain, supported by an integrated emergency care network (8–10). Access to registry data was approved by the national headquarters. This study was approved by the Ethics Committee of the Third Affiliated Hospital of Wenzhou Medical University and conducted in accordance with the principles of the Declaration of Helsinki (Ethics approval number: YSZM2025040). Owing to its retrospective design, the requirement for informed consent was waived.

### Study population

We included patients with AMI who underwent PCI for chest pain at our center between January 1, 2021, and April 2025. AMI was defined according to the Fourth Universal Definition of Myocardial Infarction (11). The inclusion criteria were as follows: (1) clinical presentation consistent with AMI; (2) electrocardiographic or biochemical evidence of myocardial infarction; (3) treatment with PCI; and (4) availability of complete data for calculating D2W time.

Exclusion criteria were as follows: (1) hospitalization for less than 1 day; (2) elective or staged PCI procedures; (3) missing data on 30-day mortality; and (4) incomplete D2W data.

### Data collection

Data were obtained from the hospital electronic medical records and through application to the headquarters of the China Chest Pain Center Information Management Platform. The China Chest Pain Center is equipped with a dedicated data registration system, in which trained personnel collect data using standardized case report forms and submit them to the central registry.

### Exposure variables

The primary exposure variable was D2W time, defined as the interval from hospital arrival (emergency department registration time) to guidewire crossing of the culprit lesion. D2W time was recorded in minutes using time stamps from the hospital registration system and catheterization laboratory logs and was verified by review of operative reports.

### Outcomes definition

The primary outcome was in-hospital mortality within 30 days of admission, defined as death from any cause within 30 days of hospital admission. Secondary outcomes included intraoperative and in-hospital complications, specifically intraoperative hypotension, malignant arrhythmia, vascular dissection, in-hospital infection, shock, bleeding, respiratory failure, and stroke.

### Covariates

Potential confounders were identified *a priori* based on clinical relevance and prior literature (12–14), including demographics, clinical presentation characteristics, cardiovascular risk factors, culprit vessel location, and initial medical therapy. The covariate sets included in each multivariable model were prespecified and are detailed in the Statistical analysis section.

### Statistical analysis

Patients were categorized into an early group (D2W ≤ 90 min) and a delayed group (D2W > 90 min). Baseline continuous variables were summarized as mean ± standard deviation or median [interquartile range (IQR)], as appropriate, and categorical variables as frequencies (percentages). Between-group comparisons were performed using the Student's t test for normally distributed continuous variables and the Wilcoxon rank-sum test for non-normally distributed continuous variables. Categorical variables were compared using the Chi-square test or Fisher's exact test, as appropriate.

For the primary outcome (in-hospital mortality within 30 days of admission), Cox proportional hazards models were used to estimate HRs and 95% confidence intervals, with D2W modeled as a continuous exposure per 10-min increase. Four prespecified models with increasing levels of adjustment were then fitted: Model 1 was unadjusted. Model 2 was adjusted for age, sex, ethnicity, history of hypertension, diabetes mellitus, hyperlipidemia, smoking, obesity, chronic obstructive pulmonary disease, and coronary artery disease. Model 3 was further adjusted for culprit vessel location, Killip class on admission, level of consciousness at admission, and emergency resuscitation measures. Model 4 was further adjusted for initial antiplatelet therapy, statin therapy, and β-blocker therapy. Time-to-event was defined as the interval from admission to death, with censoring at discharge or 30 days, whichever occurred first.

To assess potential nonlinearity, RCS terms with four knots were incorporated into the fully adjusted Cox model (Model 4) for the primary outcome and into the corresponding multivariable models for intraoperative and in-hospital complications (15). Nonlinearity was assessed using a likelihood ratio test comparing the spline model with a model including only the linear term for D2W time.

### Subgroup analyses

We repeated the RCS analyses within prespecified subgroups to evaluate the robustness of the dose–response relationship between D2W time and in-hospital mortality within 30 days of admission, as well as intraoperative and in-hospital complications. Subgroups were defined by age (<65 vs. ≥65 years), sex, diabetes status, and hypertension status.

All statistical analyses were performed using R software, version 4.5.0 (R Foundation for Statistical Computing, Vienna, Austria). A two-sided *p*-value <0.05 was considered statistically significant.

## Results

### Study population and baseline characteristics

**Figure 1:**
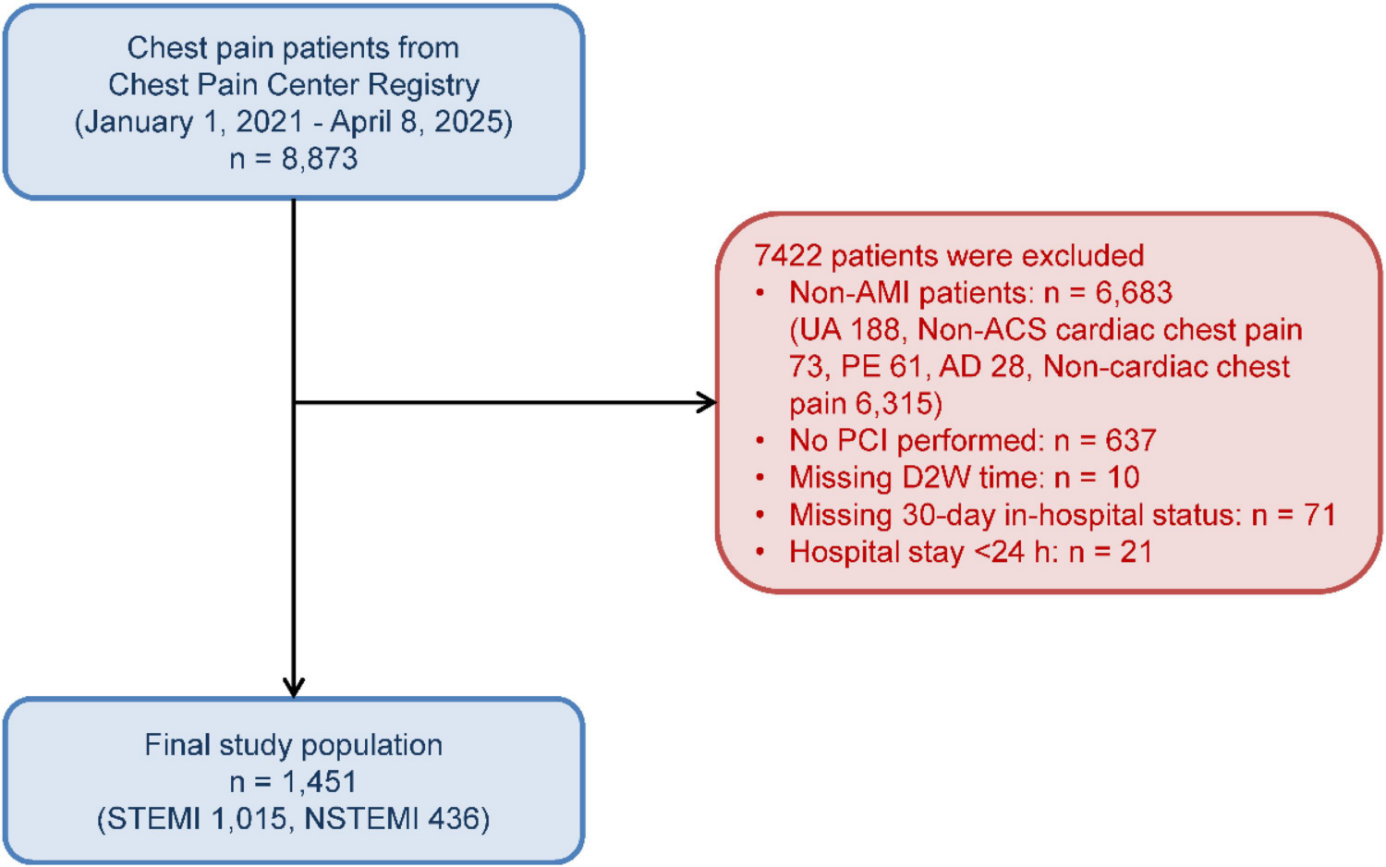
Flowchart of patient selection from the China chest pain center registry between January 1, 2021, and April 8, 2025. Exclusions were applied sequentially at each step based on the remaining cohort. AMI, acute myocardial infarction; UA, unstable angina; ACS, acute coronary syndrome; PE, pulmonary embolism; AD, aortic dissection; PCI, percutaneous coronary intervention; D2W, door-to-wire time; STEMI, ST-segment elevation myocardial infarction; NSTEMI, non–ST-segment elevation myocardial infarction.

A total of 8,873 patients with chest pain were consecutively enrolled from the China Chest Pain Center Registry between January 1, 2021, and April 8, 2025. Among them, 2,399 patients underwent PCI, of whom 22 died during hospitalization within 30 days of admission. After excluding non-AMI cases and applying the prespecified inclusion and exclusion criteria, 1,451 patients with confirmed AMI who underwent PCI were included in the final analytic cohort (Figure 1). Patients were categorized into an early group (D2W ≤ 90 min; *n* = 1,054) and a delayed group (D2W > 90 min; *n* = 397). Overall, 1,015 patients (69.9%) were diagnosed with STEMI and 436 patients (30.1%) with NSTEMI. Compared with the early group, patients in the delayed group were older and more likely to have impaired consciousness at admission, higher Killip class, hypertension, and a history of coronary artery disease; angiographic culprit-vessel distribution and initial treatments also differed between groups (Table 1).

**Table 1:** Baseline characteristics of patients with AMI undergoing PCI by D2W time (≤90 vs. >90 min).

Variables	D2W ≤ 90 (*n* = 1,054)	D2W > 90 (*n* = 397)	*P*-value
*N*	1,054	397	
Age, years, mean ± SD	59.61 ± 13.45	61.32 ± 14.76	0.032
Sex, *n* (%)			0.054
Male	894 (84.82)	320 (80.60)	
Female	160 (15.18)	77 (19.40)	
Ethnicity, *n* (%)			0.865
Han	1,038 (98.86)	391 (98.74)	
Non-Han	12 (1.14)	5 (1.26)	
Level of consciousness on admission, *n* (%)			<0.001
Unresponsive to any stimuli	12 (1.14)	13 (3.27)	
Response to pain	3 (0.28)	5 (1.26)	
Response to verbal stimuli	3 (0.28)	4 (1.01)	
Fully conscious	1,036 (98.29)	375 (94.46)	
Culprit vessel location, *n* (%)			<0.001
LAD	498 (47.25)	152 (38.29)	
RCA	356 (33.78)	112 (28.21)	
LCX	129 (12.24)	81 (20.40)	
Small artery	23 (2.18)	25 (6.30)	
LM	14 (1.33)	7 (1.76)	
LAD + LCX	15 (1.42)	14 (3.53)	
RCA + LAD	11 (1.04)	3 (0.76)	
RCA + LCX	7 (0.66)	3 (0.76)	
Killip classification on admission, *n* (%)			0.009
Class I	952 (90.32)	334 (84.13)	
Class II	14 (1.33)	6 (1.51)	
Class III	35 (3.32)	25 (6.30)	
Class IV	53 (5.03)	32 (8.06)	
AMI diagnosis, *n* (%)			<0.001
STEMI	851 (80.74)	164 (41.31)	
NSTEMI	203 (19.26)	233 (58.69)	
Medical history, *n* (%)			
Smoking status			0.011
Never	607 (57.59)	252 (63.48)	
Current	377 (35.77)	111 (27.96)	
Former	70 (6.64)	34 (8.56)	
Obesity			0.141
No	1,035 (98.20)	394 (99.24)	
Yes	19 (1.80)	3 (0.76)	
Hypertension			0.019
No	452 (42.88)	144 (36.27)	
Yes	602 (57.12)	253 (63.73)	
Diabetes mellitus			0.355
No	719 (68.22)	262 (65.99)	
Yes	335 (31.78)	135 (34.01)	
Hyperlipidemia			0.923
No	742 (70.40)	279 (70.28)	
Yes	312 (29.60)	118 (29.72)	
COPD			0.077
No	1,036 (98.29)	395 (99.50)	
Yes	18 (1.71)	2 (0.50)	
Coronary artery disease			<0.001
No	975 (92.50)	338 (85.14)	
Yes	79 (7.50)	59 (14.86)	
Initial treatment			
Antiplatelet therapy, *n* (%)			0.016
No	20 (1.90)	16 (4.03)	
Yes	1,034 (98.10)	381 (95.97)	
Statin therapy, *n* (%)			0.035
No	28 (2.66)	18 (4.53)	
Yes	1,024 (97.34)	379 (95.47)	
β-blocker therapy, *n* (%)			0.318
No	1,034 (98.10)	387 (97.48)	
Yes	20 (1.90)	10 (2.52)	
Primary outcome			
In-hospital death within 30 days, *n* (%)			0.005
No	1,054 (100.00)	394 (99.24)	
Yes	0 (0.00)	3 (0.76)	
Secondary outcomes			
Intraoperative complication			
Hypotension, *n* (%)			0.247
No	943 (89.64)	363 (91.67)	
Yes	109 (10.36)	33 (8.33)	
Arrhythmia, *n* (%)			0.002
No	1,002 (95.25)	391 (98.74)	
Yes	50 (4.75)	5 (1.26)	
Vascular dissection (Aortic), *n* (%)			0.146
No	1,041 (98.95)	388 (97.98)	
Yes	11 (1.05)	8 (2.02)	
In-hospital complication			
Infection, *n* (%)			<0.001
No	973 (92.40)	338 (85.14)	
Yes	80 (7.60)	59 (14.86)	
Shock, *n* (%)			0.001
No	1,016 (96.49)	367 (92.44)	
Yes	37 (3.51)	30 (7.56)	
Bleeding, *n* (%)			0.113
No	1,039 (98.67)	387 (97.48)	
Yes	14 (1.33)	10 (2.52)	
Respiratory failure, *n* (%)			<0.001
No	1,031 (97.91)	368 (92.70)	
Yes	22 (2.09)	29 (7.30)	
Stroke, *n* (%)			0.177
No	1,047 (99.43)	392 (98.74)	
Yes	6 (0.57)	5 (1.26)	

Percentages were calculated based on non-missing data.

Values are presented as mean ± standard deviation (SD) for continuous variables and *n* (%) for categorical variables. *P* values were calculated using the Student's t test or Mann–Whitney U test for continuous variables, and the *χ*^2^ test or Fisher's exact test for categorical variables, as appropriate. In the overall cohort (*N* = 1,451), 1,015 (70.0%) were diagnosed with STEMI and 436 (30.0%) with NSTEMI.

D2W, door-to-wire time; SD, standard deviation; LAD, left anterior descending artery; RCA, right coronary artery; LCX, left circumflex artery; LM, left main coronary artery; COPD, chronic obstructive pulmonary disease; STEMI, ST-segment elevation myocardial infarction; NSTEMI, non–ST-segment elevation myocardial infarction.

### Nonlinear associations of D2W time with 30-day in-hospital mortality and complications based on restricted cubic spline models

**Figure 2:**
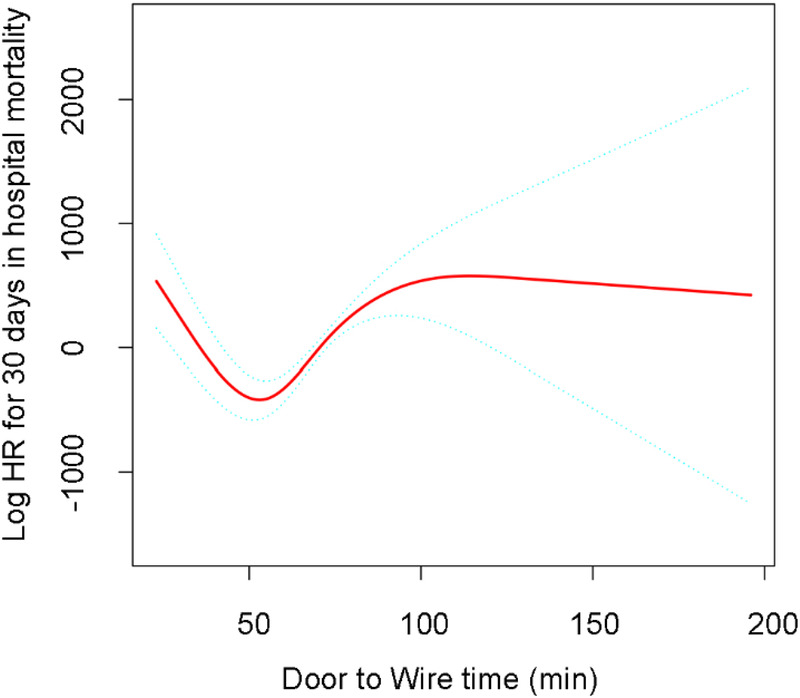
Association between door to wire time and 30-day in-hospital mortality. Restricted cubic spline analysis was performed with D2W time modeled continuously using the fully adjusted Cox model (Model 4). The model was adjusted for age, sex, ethnicity, history of hypertension, diabetes mellitus, hyperlipidemia, smoking, obesity, chronic obstructive pulmonary disease, coronary artery disease, culprit vessel location, Killip class on admission, level of consciousness at admission, emergency resuscitation measures, and initial antiplatelet therapy, statin therapy, and β-blocker therapy. The solid line represents the estimated log hazard ratio, and the dashed lines represent the 95% confidence intervals.

**Figure 3:**
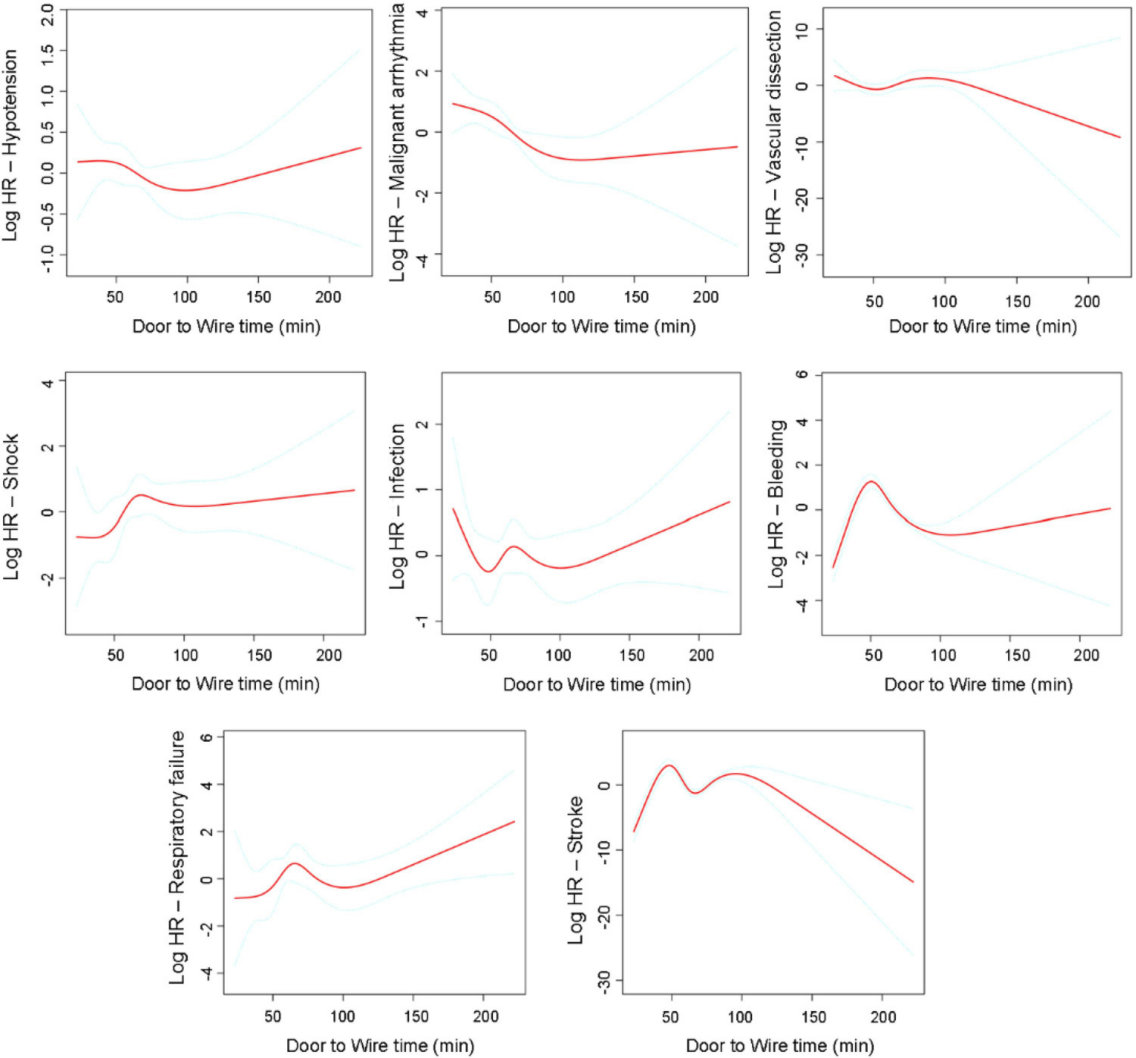
Association between door to wire time and the risk of intraoperative and in-hospital complications. Restricted cubic spline analyses were performed with D2W time modeled continuously using multivariable models adjusted for the same covariates as the fully adjusted model for the primary outcome (Model 4): age, sex, ethnicity, history of hypertension, diabetes mellitus, hyperlipidemia, smoking, obesity, chronic obstructive pulmonary disease, coronary artery disease, culprit vessel location, Killip class on admission, level of consciousness at admission, emergency resuscitation measures, and initial antiplatelet therapy, statin therapy, and β-blocker therapy. Solid lines represent the estimated log hazard ratios, and dashed lines represent the 95% confidence intervals.

In multivariable Cox models, each 10-min increase in D2W time was associated with a higher risk of in-hospital mortality within 30 days of admission in the fully adjusted model (HR 1.47, 95% CI 1.16–1.87; *P* = 0.0016) (Table 2). In the multivariable-adjusted RCS analyses, the associations between D2W time and in-hospital mortality within 30 days of admission suggested a non-linear pattern (Figure 2). The spline curve indicated that the estimated log(HR) declined during the early period, reached a minimum at approximately 50 min, and then increased gradually as D2W time lengthened, with the association plateauing at longer delays. Regarding complications, longer D2W time was generally associated with higher risks of in-hospital infection and respiratory failure, whereas associations with intraoperative hypotension, malignant arrhythmia, vascular dissection, shock, bleeding, and stroke were weaker and more imprecise (Figure 3).

**Table 2:** Association between D2W time and in-hospital mortality within 30 days of admission.

Exposure	Model 1 HR (95% CI) *P* value	Model 2 HR (95% CI) *P* value	Model 3 HR (95% CI) *P* value	Model 4 HR (95% CI) *P* value
D2W (per 10 min)	1.04 (0.95–1.13) 0.3828	1.05 (0.95, 1.17) 0.3529	1.44 (1.12, 1.84) 0.0038	1.47 (1.16–1.87) 0.0016

D2W was modeled per 10-min increase.

Model 1 was unadjusted.

Model 2 was adjusted for age, sex, ethnicity, history of hypertension, diabetes mellitus, hyperlipidemia, smoking, obesity, chronic obstructive pulmonary disease, and coronary artery disease.

Model 3 was further adjusted for culprit vessel location, Killip class on admission, level of consciousness at admission, emergency resuscitation measures.

Model 4 was further adjusted for initial antiplatelet therapy, statin therapy, and β-blocker therapy.

### Subgroup analyses

RCS analyses repeated in prespecified subgroups (age, sex, diabetes status, and hypertension status) showed patterns broadly consistent with the primary results (Supplementary Figure S1–S8). Across subgroups, the curves indicated lower mortality at shorter D2W times, with the estimated hazard ratio (HR) reaching its minimum at approximately 50 min, followed by a gradual increase as D2W time lengthened. Associations with infection and respiratory failure were directionally consistent across subgroups.

## Discussion

In this single-center, registry-based study of patients with AMI undergoing PCI at a certified Chest Pain Center, we evaluated the association between D2W time and short-term outcomes. In multivariable analyses, longer D2W time was associated with a higher risk of in-hospital mortality within 30 days of admission. RCS analyses further suggested a nonlinear association, with the estimated HR lowest at approximately 50 min and increasing gradually as D2W time lengthened. Longer D2W time was also associated with higher risks of in-hospital infection and respiratory failure.

To our knowledge, this is the first study to analyze D2W time as a continuous variable in relation to 30-day in-hospital mortality and to examine its dose–response relationship. Our findings align with prior evidence (5, 16) that shorter reperfusion delays are associated with better outcomes in AMI. However, the existing literature and performance efforts have largely focused on door-to-balloon time and broader system-delay metrics (3, 4, 17, 18), whereas evidence specifically addressing D2W time remains limited. By modeling D2W time continuously and characterizing the dose–response relationship, our study provides clinically relevant information beyond a single dichotomous threshold.

Several studies have examined outcomes in STEMI patients undergoing primary PCI by contrasting door-to-balloon times ≤90 min with longer delays. In National Registry of Myocardial Infarction data, McNamara et al. (19) reported a graded increase in in-hospital mortality across D2B categories, with higher adjusted mortality when D2B exceeded 90 min. Using a large national cohort, Rathore et al. (20) demonstrated that the D2B–mortality relationship is continuous and non-linear, with risk rising progressively as delays lengthen rather than reflecting a simple threshold effect. In contrast, several temporal trend studies observed that substantial improvements in D2B performance at the population level were not accompanied by proportional reductions in mortality. For example, Menees et al. (17) reported marked improvements in D2B times and increasing proportions achieving ≤90 min, yet no significant overall change in in-hospital or 30-day mortality over time. Similarly, Flynn et al. (21) found dramatic decreases in median D2B time and improved compliance with the 90-minute benchmark, while in-hospital mortality remained essentially unchanged. To explain this apparent inconsistency, we drew on the interpretation proposed by Nallamothu et al. (22). Although shorter D2B time remains associated with a lower risk of death at the individual level, changes in case mix and contemporaneous advances in treatment and system-wide care capabilities at the population level may attenuate the statistical detectability of this mortality benefit. Consistent with the individual-level D2B literature (19, 20), we observed that longer in-hospital reperfusion delay was associated with worse short-term outcomes. However, rather than focusing on D2B, we evaluated D2W time in an AMI cohort (including both STEMI and NSTEMI) undergoing PCI, modeled D2W as a continuous exposure, and identified a non-linear association with short-term outcomes.

A key observation was the non-linear relationship between D2W time and 30-day in-hospital mortality, with the fitted spline curve reaching its lowest point at approximately 50 min. Importantly, this time point should not be interpreted as a “target” that is universally optimal. Instead, it likely reflects the lowest point of the fitted curve in our dataset and may be influenced by workflow efficiency and case mix. In routine practice, patients with very short D2W times may include those with greater initial severity who are triaged urgently, whereas modest delays may occur in more stable patients. Longer delays may also reflect diagnostic uncertainty, system congestion, delayed catheter-lab activation, or the need for stabilization before PCI. Together, these factors may contribute to a U-shaped or J-shaped association. Clinically, the main implication is that longer in-hospital process delays, reflected by prolonged D2W time, are associated with worse short-term outcomes rather than that care should aim for a specific minute value.

We also found that longer D2W time was associated with in-hospital infection and respiratory failure. One plausible explanation is that delayed reperfusion may reflect a greater ischemic burden and contribute to more extensive myocardial injury, leading to early hemodynamic compromise (e.g., pulmonary congestion/edema or cardiogenic shock) and a higher likelihood of requiring intensive care and invasive respiratory support such as mechanical ventilation (23, 24). These factors can predispose patients to respiratory failure and simultaneously increase exposure to hospital-acquired infections through prolonged ICU stay and the use of invasive devices (e.g., endotracheal intubation and intravascular/urinary catheters) (25, 26). These associations were directionally consistent in subgroup analyses.

This study has several strengths. First, we leveraged the standardized data infrastructure of China Chest Pain Center. Each participating site employs trained personnel for real-time case registration, and data are reported through a dedicated platform that captures the full clinical course—from symptom onset to discharge. This system ensures comprehensive documentation of clinical characteristics, medical history, treatment strategies, and intraoperative and in-hospital complications, thereby supporting both the completeness and accuracy of the dataset. Second, we used complementary analytic approaches: multivariable Cox models quantified the overall association, RCS modeling characterized potential nonlinearity. Third, we repeated RCS analyses in prespecified subgroups to assess robustness of the dose–response relationship.

Several limitations should be noted. First, because this was a retrospective, single-center study of AMI patients undergoing PCI (including both STEMI and NSTEMI), generalizability may be limited and residual confounding cannot be ruled out despite multivariable adjustment and sensitivity analyses. Second, in-hospital death within 30 days was relatively infrequent in this AMI cohort, which may affect the robustness of our conclusions. We therefore performed multiple prespecified sensitivity analyses. The spline shape, including the apparent minimum around 50 min, should be interpreted with caution. Third, D2W time may be prone to measurement error and documentation variability. In addition, we captured only in-hospital mortality within 30 days of admission and could not account for out-of-hospital deaths after discharge. Finally, we evaluated multiple secondary outcomes. Although clinically motivated, these analyses are susceptible to chance findings and should be confirmed in larger cohorts, preferably in prospective studies with prespecified endpoints.

## Conclusion

In conclusion, in this single-center Chest Pain Center cohort, longer D2W time was associated with higher in-hospital mortality within 30 days of admission and with higher likelihood of infection and respiratory failure. The association between D2W time and 30-day mortality was non-linear, with the lowest estimated risk around 50 min and increasing with longer delays. Further multicenter studies are warranted to validate these findings.

## Data Availability

The raw data supporting the conclusions of this article will be made available by the authors, without undue reservation.
