# Supplementary material for: Association between door-to-wire time and 30-day mortality after PCI in patients with acute myocardial infarction: evidence from a single-center study in the China chest pain center registry
Source: Front Cardiovasc Med. 2026 Feb 18;13:1717258. doi: 10.3389/fcvm.2026.1717258 (PMC12957179; doi:10.3389/fcvm.2026.1717258)
Supplement: Supplementary file 1 [file Datasheet1.pdf]

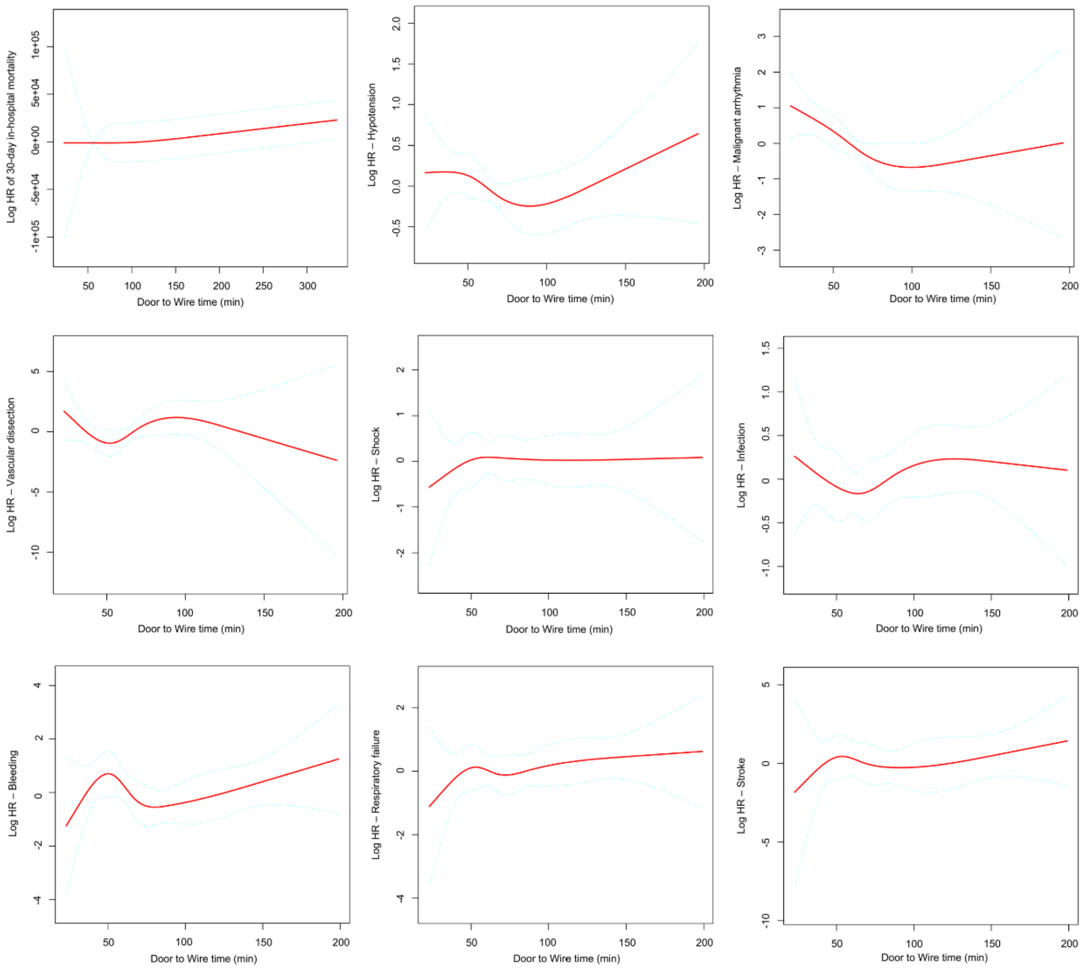


**Figure S1.** Restricted cubic spline analysis of the association between door-to-wire time and 30-day mortality and complications in male patients.

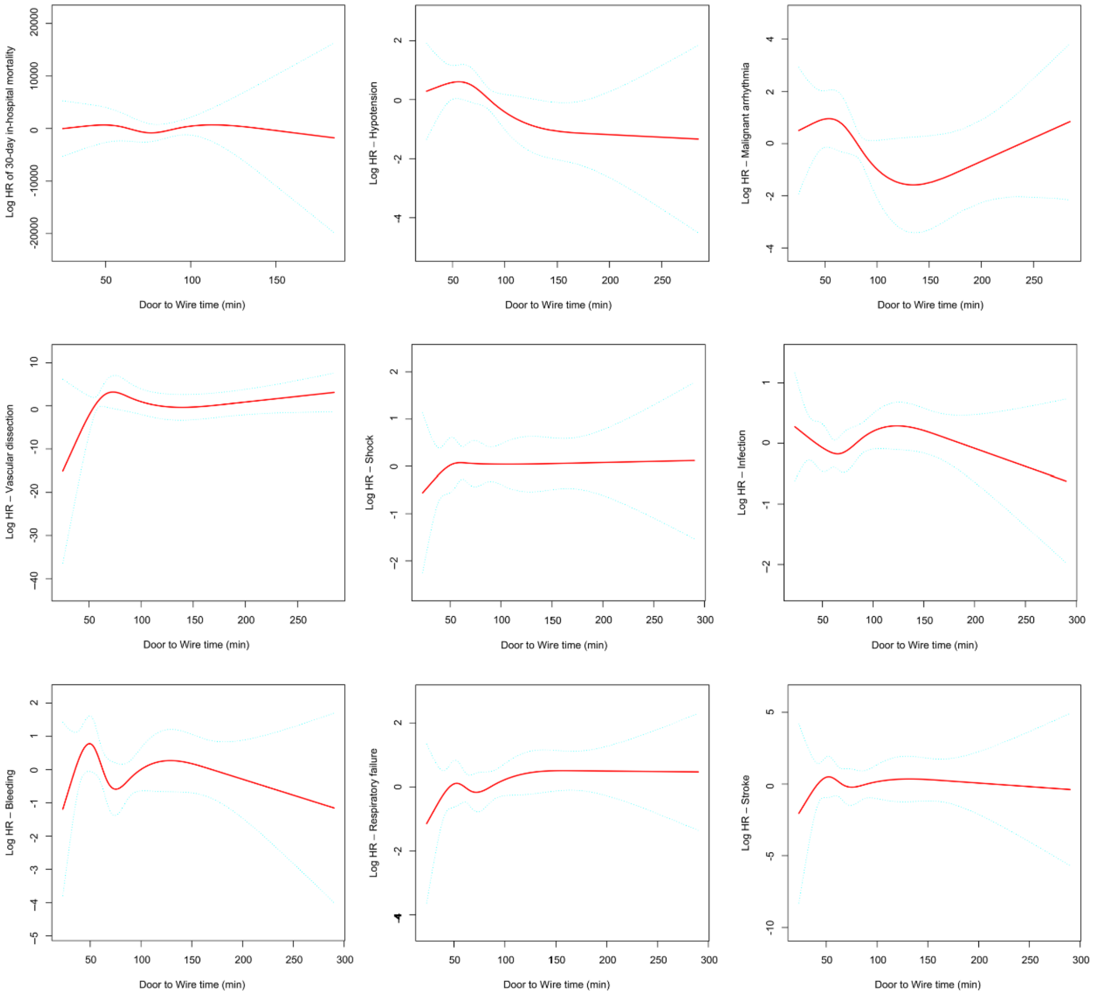


**Figure S2.** RCS analysis of the association between D2W time and 30-day mortality and complications in female patients.

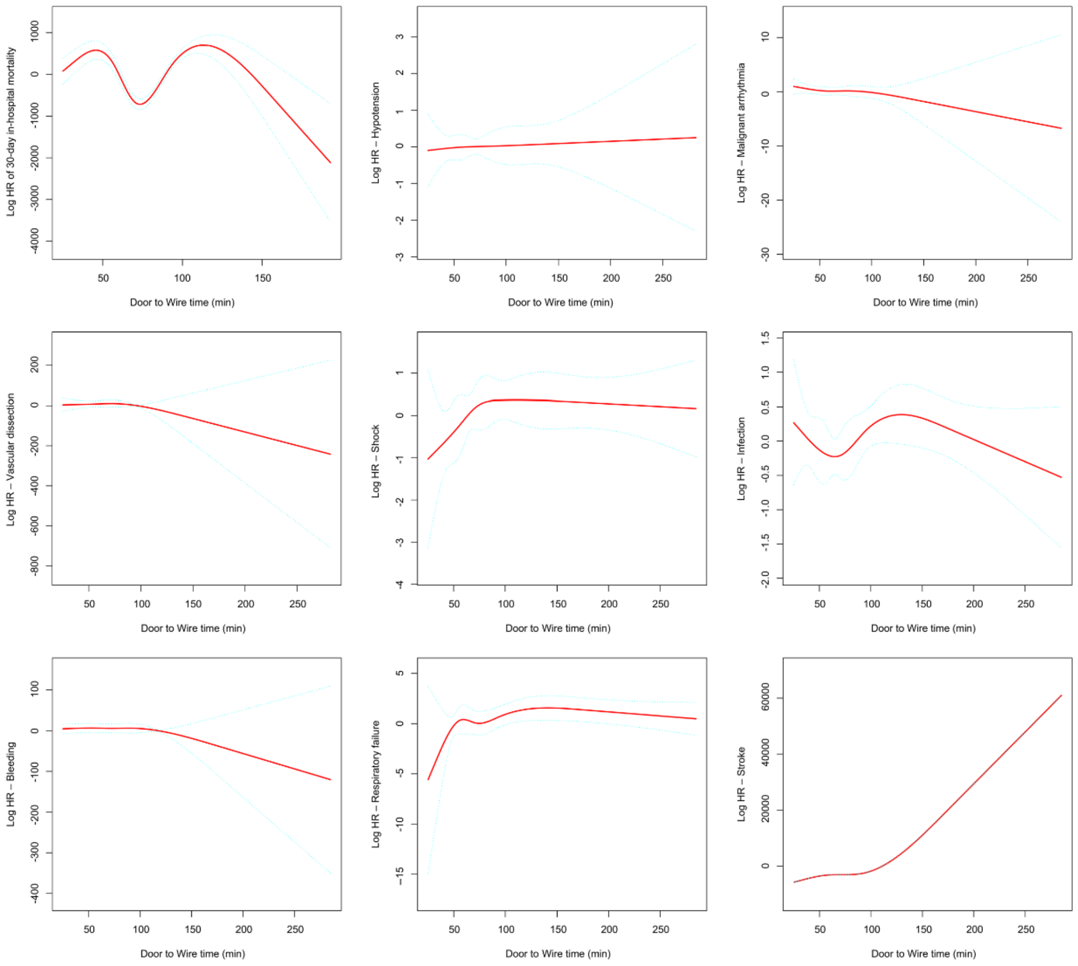


**Figure S3.** RCS analysis of the association between D2W time and 30-day mortality and complications in patients aged up 65 years.

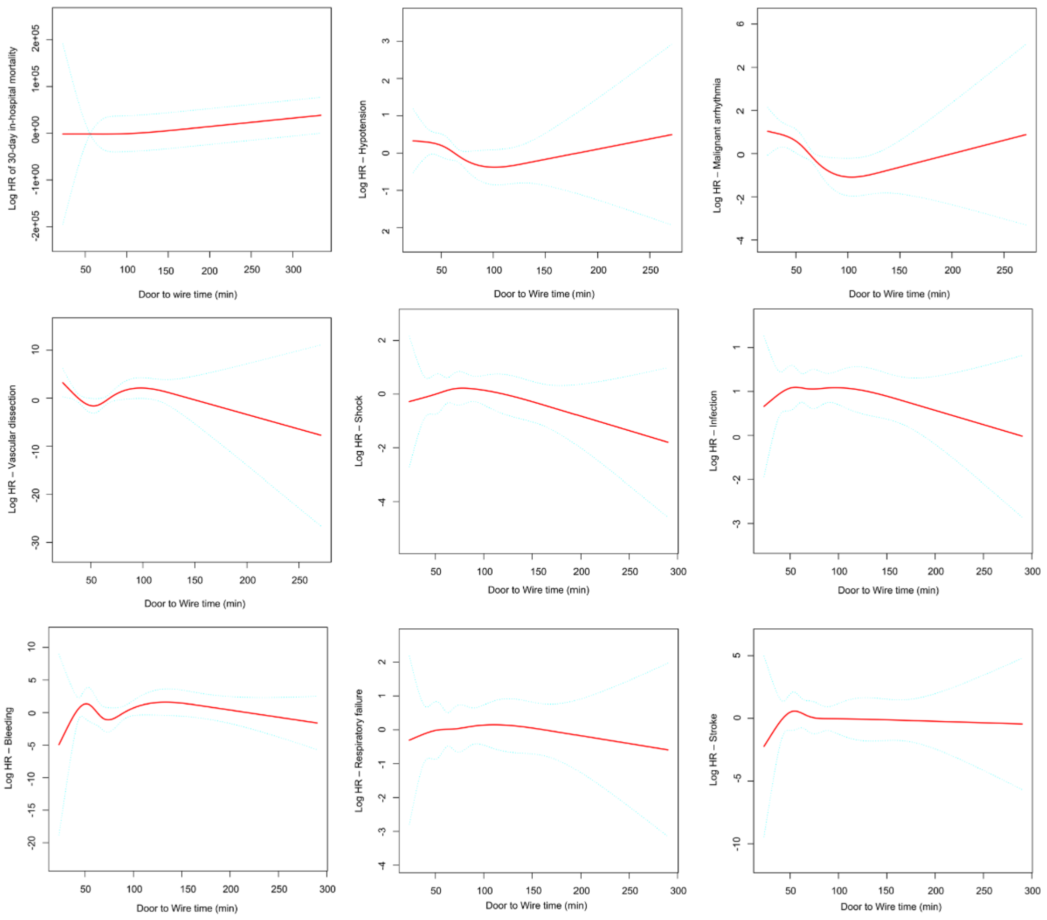


**Figure S4.** RCS analysis of the association between D2W time and 30-day mortality and complications in patients aged below 65 years.

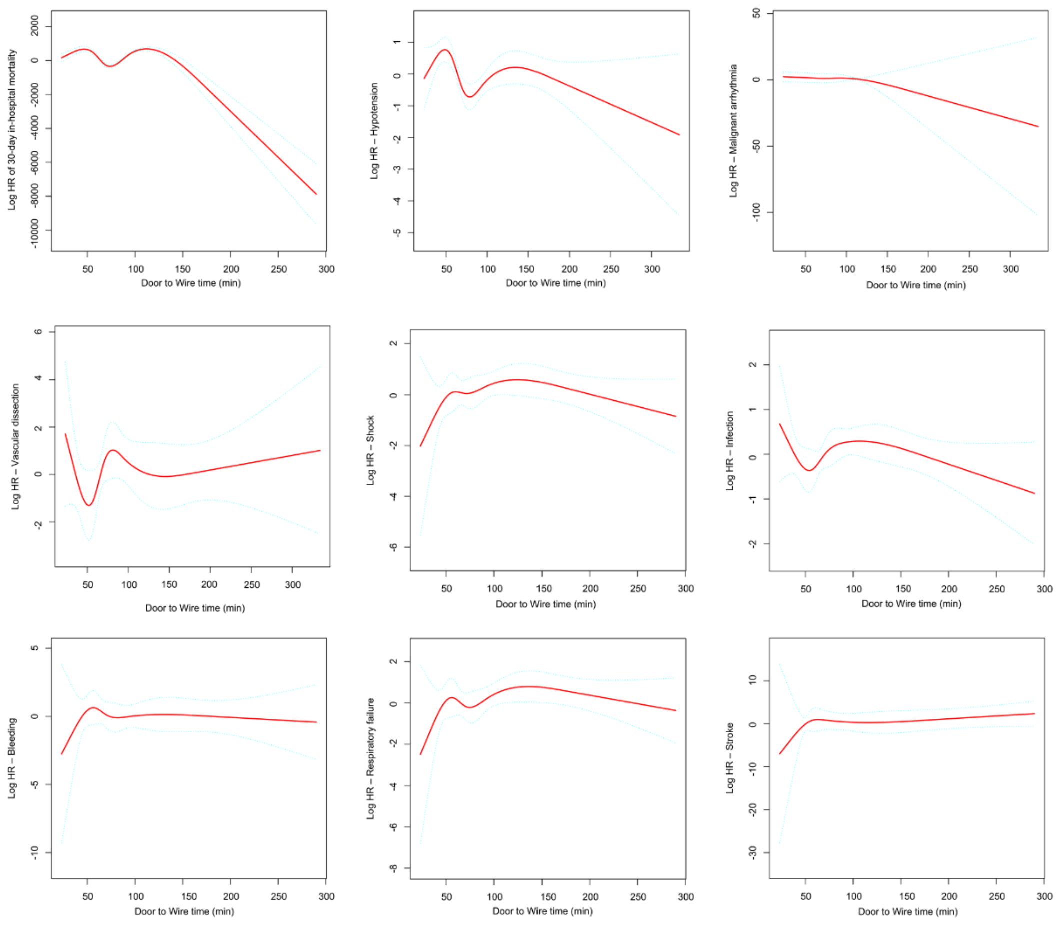


**Figure S5.** RCS analysis of the association between D2W time and 30-day mortality and complications in patients with hypertension.

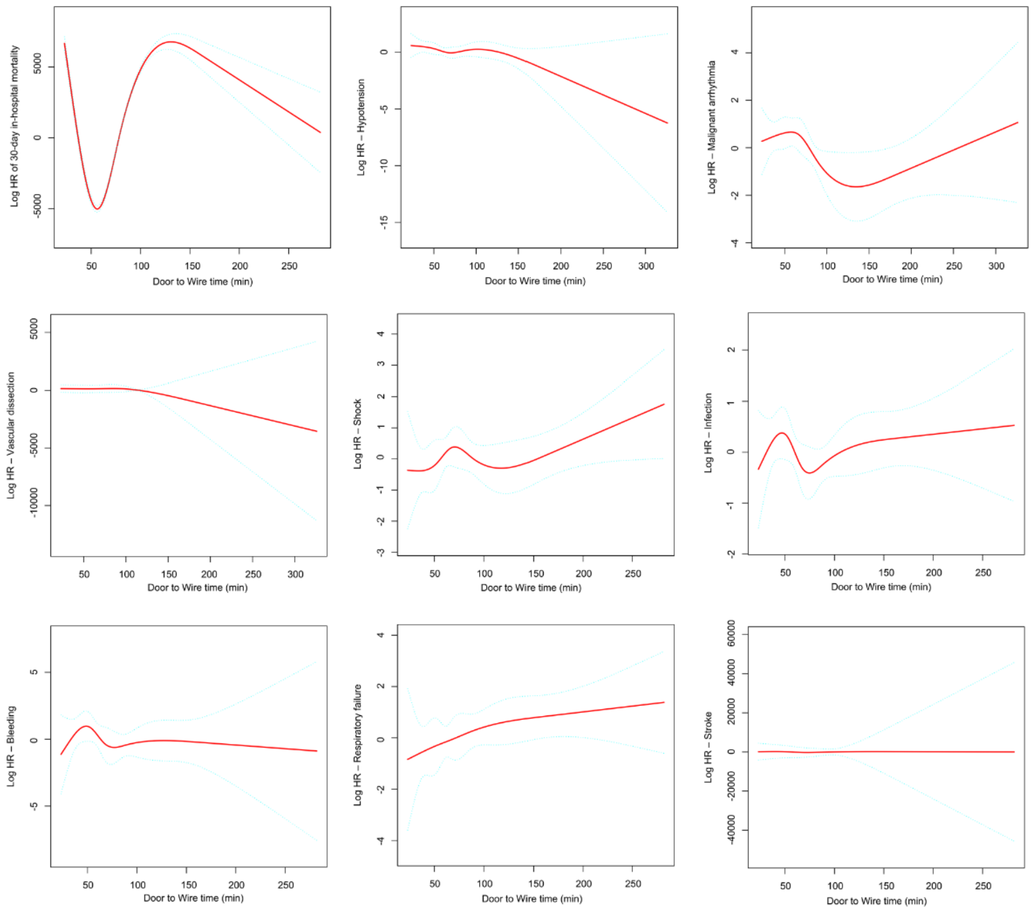


**Figure S6.** RCS analysis of the association between D2W time and 30-day mortality and complications in patients without hypertension.

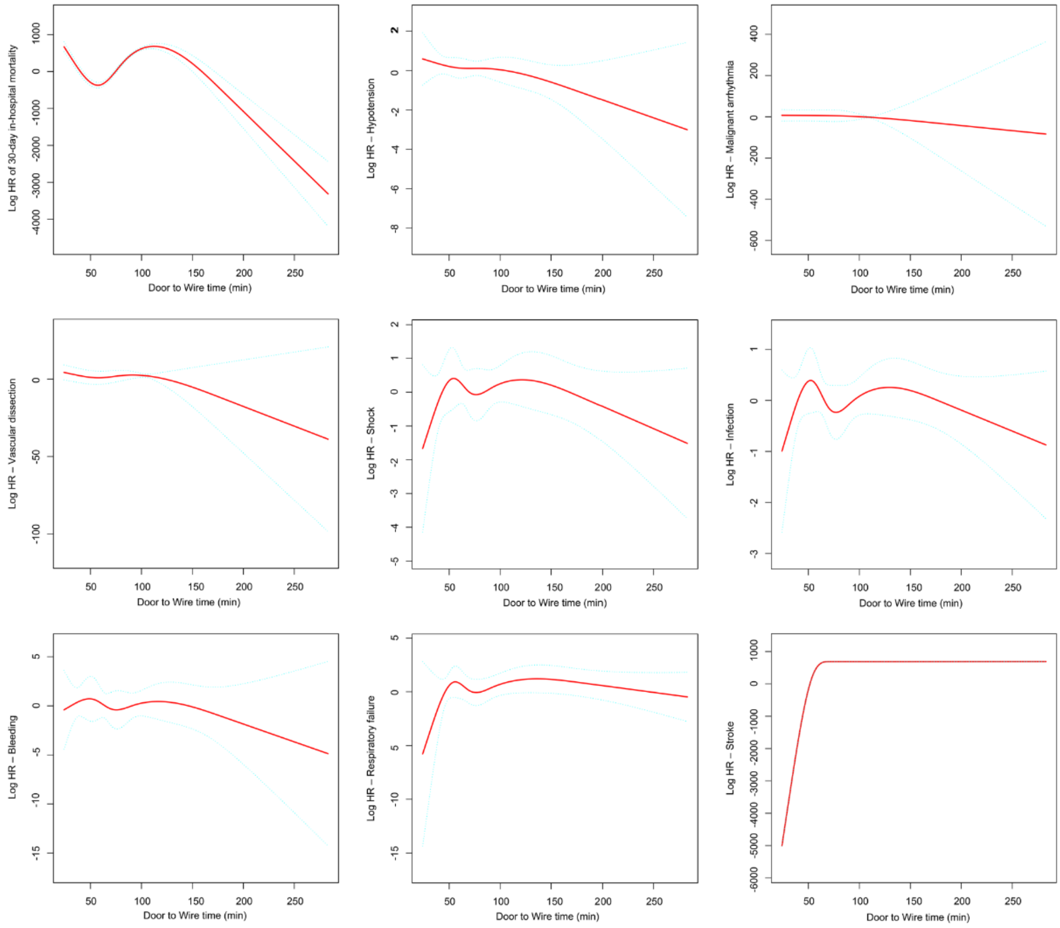


**Figure S7.** RCS analysis of the association between D2W time and 30-day mortality and complications in patients with diabetes.

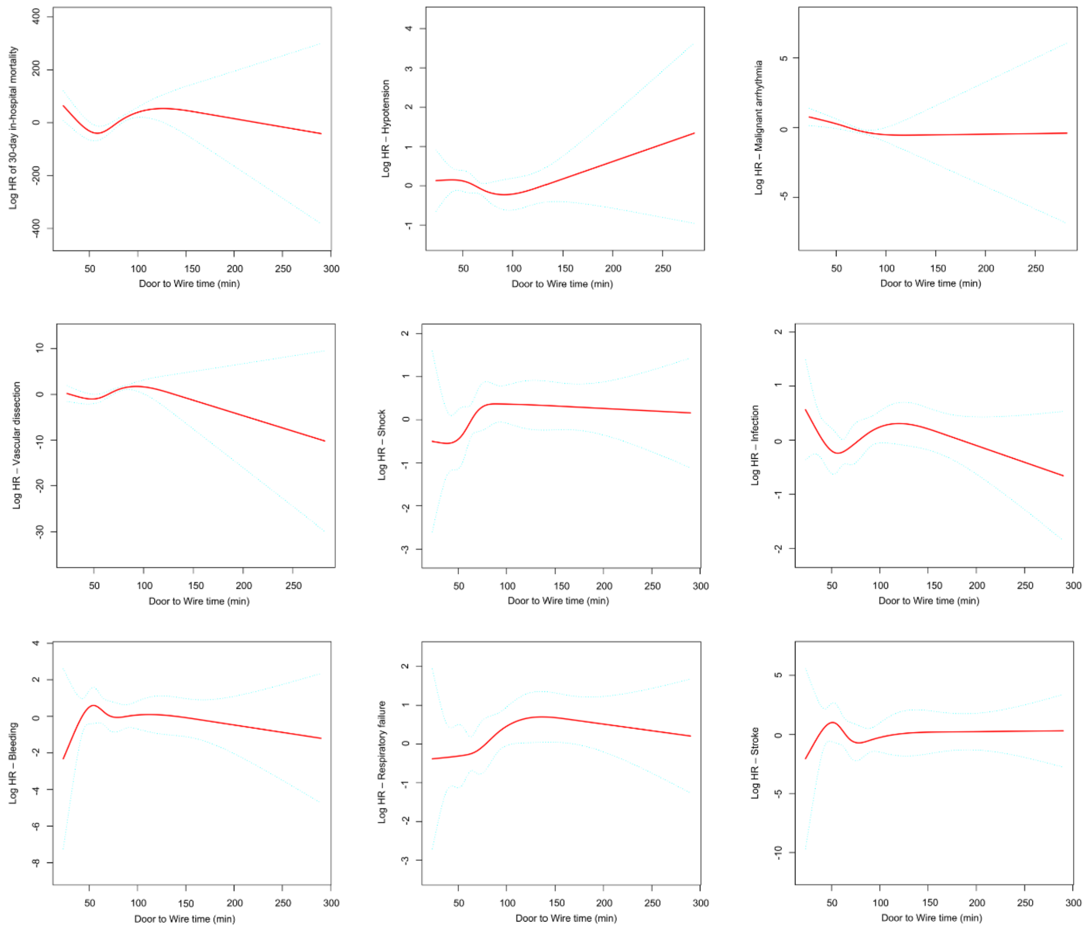


**Figure S8.** RCS analysis of the association between D2W time and 30-day mortality and complications in patients without diabetes.
